# Short-Term Diet Restriction but Not Alternate Day Fasting Prevents Cisplatin-Induced Nephrotoxicity in Mice

**DOI:** 10.3390/biomedicines8020023

**Published:** 2020-02-03

**Authors:** Evrin Gunebakan, Esra Yalcin, Esra Cikler Dulger, Ahmet Yigitbasi, Nilay Ates, Aysun Caglayan, Mustafa C. Beker, Kazim Sahin, Hasan Korkaya, Ertugrul Kilic

**Affiliations:** 1 Department of Physiology, School of Medicine, Istanbul Medipol University, Istanbul 34810, Turkey; evrin89e@gmail.com (E.G.); esraylcn@gmail.com (E.Y.); acaglayan@medipol.edu.tr (A.C.); mcbeker@medipol.edu.tr (M.C.B.); 2 Department of Histology and Embryology, Hamidiye Medical School, University of Health Sciences, Istanbul 34668, Turkey; esracikler@gmail.com; 3 Department of Internal Medicine, School of Medicine, Trakya University, Edirne 22030, Turkey; ahmetyigitbasi23@gmail.com; 4 Department of Pharmacology, School of Medicine, Istanbul Medipol University, Istanbul 34810, Turkey; nates@medipol.edu.tr; 5 Animal Nutrition Department, School of Veterinary Medicine, Firat University, Elazig 23119, Turkey; nsahinkm@yahoo.com; 6 Department of Biochemistry and Molecular Biology, Medical College of Georgia, Augusta University, Augusta, GA 30912, USA; hkorkaya@augusta.edu

**Keywords:** cisplatin, fasting, renal protection, acute kidney injury, nephrotoxicity, MAPK pathway

## Abstract

Cisplatin (CP) is one of the most preferred platinum-containing antineoplastic drugs. However, even in nontoxic plasma concentrations, it may cause kidney injury. To be able to increase its effective pharmacological dose, its side effects need to be regarded. Diet restriction (DR) has been demonstrated to improve cellular survival in a number of disorders. In this context, we investigated the role of DR in CP-induced nephrotoxicity (CPN). Besides alternate DR, animals were exposed to DR for 3 days prior or after CP treatment. Here, we observed that both 3 days of DR reverses the nephrotoxic effect of CP, which was associated with improved physiological outcomes, such as serum creatine, blood-urea nitrogen and urea. These treatments significantly increased phosphorylation of survival kinases PI3K/Akt and ERK-1/2 and decreased the level of stress kinase JNK were noted. In addition, the activation level of signal transduction mediator p38 MAPK phosphorylation was higher particularly in both three-day DR groups. Next, animals were fed with carbohydrate-, protein- or fat-enriched diets in the presence of CP. Results indicated that not only fasting but also dietary content itself may play a determinant role in the severity of CPN. Our data suggest that DR is a promising approach to reduce CPN by regulating metabolism and cell signaling pathways.

## 1. Introduction

Cisplatin (Cis-diaminedichloroplatinum, CDDP or CP) is the most commonly used platinum-containing antineoplastic drug for the treatments of various solid tumors, ranging from bladder [1] to breast [2] to non-small cell lung cancer [3]. It may cause serious parenchymal toxicity due to its accumulation in the kidney tissue, as it is usually excreted in the urine. Even though cisplatin-induced nephrotoxicity (CPN) could be alleviated by diuretics, prevalence of CPN is still high [4]. Approximately in 60% of CP-treated patients, acute kidney injury (AKI) is developed, in which renal vasoconstriction, decrease in glomerular filtration rate and increase in serum creatinine levels were observed [5]. This dose-limiting side effect of CP renders dose augmentation almost impossible in patients requiring higher therapeutic efficacy. 

Fasting or diet restriction (DR) is described as food deprivation from the system for certain periods of time [6]. Several lines of clinical and nonclinical studies suggested that DR prior to chemotherapy potentiates the effect of the treatment by sensitizing cancer cells to chemotherapy, through a decrease in circulating growth factors and by supporting healthy cells to become more resistance to oxidative stress [7,8,9,10]. Alternate day fasting (AF) is one of the experimental DR models in which 24 hour long cycles of feeding and fasting are applied. In AF, food is provided ad libitum for 24 h, followed by a 24 h long period of fasting (no food or reduced food) [11]. Studies have shown that AF has antineoplastic effects, such as inhibition of tumor growth and tumor cell proliferation, and decreased insulin-like growth factor-1 (IGF-1) levels [12,13,14,15,16,17]. In addition, prolonged fasting (PF) lasting for 48–120 h has been studied in terms of cellular survival and antineoplastic benefits. The full switch to fat and ketone bodies in prolonged fasting conditions enhances tumor cells’ resistance to toxicities in both mice and humans and inhibits tumor growth [18,19]. It has also been shown that PF can play a role in chemoprotection and promote hematopoietic stem-cell self-renewal and reconstitution [18].

However, the cell-protective effect of DR on CPN is largely unknown. In this study, we investigated the effect of AF or fasting for 72 h prior to or after CP-treatment on CPN and analyzed renal functions and cellular survival using histological examinations. Next, we evaluated the activations of signaling pathways, including survival and stress kinases. Finally, to further identify the effect of dietary contents, we compared the nephrotic histopathologies in ad libitum feeding and protein-, carbohydrate- or lipid-rich diet groups.

## 2. Materials and Methods

### 2.1. Animals

Adult male Balb/c mice (*n* = 60, 14–17 weeks old) weighing 30 ± 4 g were kept in an environmentally controlled room with a constant temperature and humidity. Animals were maintained into a 12:12 h long light–dark cycle and had free access to water and standard rodent diet ad libitum. All experimental procedures were carried out with local governmental approval (Istanbul Medipol University, 29/8/2014), according to NIH guidelines for the care and use of laboratory animals

### 2.2. Experimental Design

In the first set of experiments, animals were randomly assigned to one of 4 groups (*n* = 8/ group/ each), with continuous water accessibility. Then, 7 mg/kg of cisplatin (CP) (Sigma Chemical Co, MO, USA) dissolved in 0.9% saline (1 mL/100 mg) [20,21] was administered, with a single intraperitoneal (i.p.) injection for the induction of CP- induced nephrotoxicity (CPN). (i) Control group with ad libitum feeding; (ii) alternate day fasting (AF) group were maintained in a eating-fasting cycle for 14 days with 24 h ad libitum feeding, followed by 24 h fasting period with CP injection being done on day 7; (iii) 72P group fasted for 72 h before CP injection; (iv) 72A group fasted for 72 after CP injection. All mice were sacrificed by using high dose chloralhydrate anesthesia one week after CP administration (day 14). Blood was collected, and kidneys were removed and shock-frozen on dry ice.

In the second set of experiments, animals were divided into 4 groups (*n* = 7/group/ each) and treated with isocaloric diets. (i) Control group received ad libitum feeding (Altromin); (ii) carbohydrate group was fed with 79.6 % carbohydrates, 10.6% fat and 9.8% protein; (iii) protein group was fed with 76.9% protein, 11.7% carbohydrates and 11.3% fat; (iv) fat group was fed with 78.4% fat, 11.2% carbohydrates and 10.4% protein. Feeding started on day 0, cisplatin (7 mg/kg) was administered on day 7 and animals were sacrificed on day 10. 

### 2.3. Histopathological Analysis

Two sets of 6 µm thick frozen cryostat (LEICA CM 1950, Wetzlar, Germany) sections from each animal were prepared for histopathological examinations. Both sets were fixed in 70% alcohol and stained according to standard protocols. One set was stained with hematoxylin and eosin (H&E) for the general morphologic examinations, and the others were stained with periodic acid–Schiff (PAS) for the examination of glomerular constitution, brush border of the differentiated apical surface of proximal tubular epithelial cells and basal membranes. Histomorphometric evaluation of H&E stained cortical and cortico-medullary proximal tubules was conducted based on acute tubular necrosis criteria, including tubular epithelia loss, intercellular cavities, brush-border loss, swollen apical membranes [22,23] and acute tubular damage criteria, including epithelia loss, pallid/pale stained cytoplasm and vacuolization, brush border loss and luminal dilatation caused by thinning tubule epithelia [22].

For each kidney section, 5 light microscopic fields at a magnitude of ×200 were examined. Distribution and existence of lesions mentioned above were evaluated on a scale of 0–4. Microscopic areas showing no damage were scored 0; microscopic areas showing damage less than 10% were scored 1; between 10–25% were scored 2; between 26–75% were scored 3; and between 76–100% were scored 4 [23,24].

### 2.4. Biochemical Analysis

Blood samples were collected and centrifuged at 10,000 rpm for 10 min at room temperature (22 °C), and sera were collected and stored at −80 °C for subsequent measurements of renal functions. Serum creatine, blood urea nitrogen and urea levels were measured with the Roche (Basel, Switzerland) C501 instrument, photometrically with the instruction kits, according to the manufacturer’s protocol.

### 2.5. Western Blot

Tissue samples belonging to the same group were pooled, homogenized and treated with protease and phosphatase inhibitor cocktails. Protein concentrations were measured with Qubit 2.0 Fluorimeter, according to the manufacturer’s protocol (Invitrogen, CA, USA). Equal amounts of protein (20 μg) were loaded on Any kD™ Mini-PROTEAN gels and run at 100 V for 2 h. Size-fractioned proteins were then transferred to polyvinylidene fluoride membrane (PVDF), using a semidry Turbo Transfer System (Bio-Rad, CA, USA). Membranes were blocked in 5% nonfat milk in 50 mM of Tris-buffered saline containing 0.1% Tween (blocking solution), for 1 h, washed in Tris-buffered saline containing 0.1% Tween (TBS-T) and incubated overnight with rabbit anti-phospho Akt (4060; Cell Signaling Technology, MA, USA), rabbit anti-total Akt (9272; Cell Signaling), rabbit anti-phospho ERK-1/-2 (9101; Cell Signaling), rabbit anti-total ERK-1/2 (9102; Cell Signaling), mouse anti-phospho JNK-1/2 (9255; Cell Signaling), rabbit anti-total JNK-1/2 (9252; Cell Signaling), rabbit anti-phospho p38 (9211; Cell Signaling) and rabbit anti-total p38 (9212; Cell Signaling). Each antibody was diluted 1:1000 directly in blocking solution. On the following day, membranes were incubated with peroxidase-conjugated goat anti-rabbit (Amersham, GE Health Care, MA, USA) or goat anti-mouse (7076; Cell Signaling) antibodies. Protein loading was controlled by stripping and re-probing the blots with total antibodies for each phospho antibody of interest. Blots were developed with Bio Rad ECL kit (Clarity™ Western ECL Substrate, #1705060) and visualized by the ChemiDoc MP (Biorad, CA, USA) system. The intensity of each signal was measured on a total of three digitized blots each, using the ImageJ software program. Protein levels were analyzed densitometrically and corrected with their total proteins each and expressed as a percent of total protein.

### 2.6. Statistical Analysis

Statistical analysis for the histologic score values was performed by using the Kruskal–Wallis test and Dunn′s multiple comparison test for the histopathological score values and group comparison, respectively. Data comparisons of Western blot and, biochemical analyses of serum samples were analyzed with a standard software package (SPSS 18 for Windows; SPSS Inc., Chicago, IL, USA). Differences between groups were analyzed by one-way ANOVA, followed by LSD test. All values were given as mean ± S.D., with n values, indicating the number of animals/each group analyzed. The *p*-values < 0.05 are considered significant.

## 3. Results

### 3.1. Prolonged Starvation Resulted in Conserved Morphology in Nephrotic Structures

**Figure 1:**
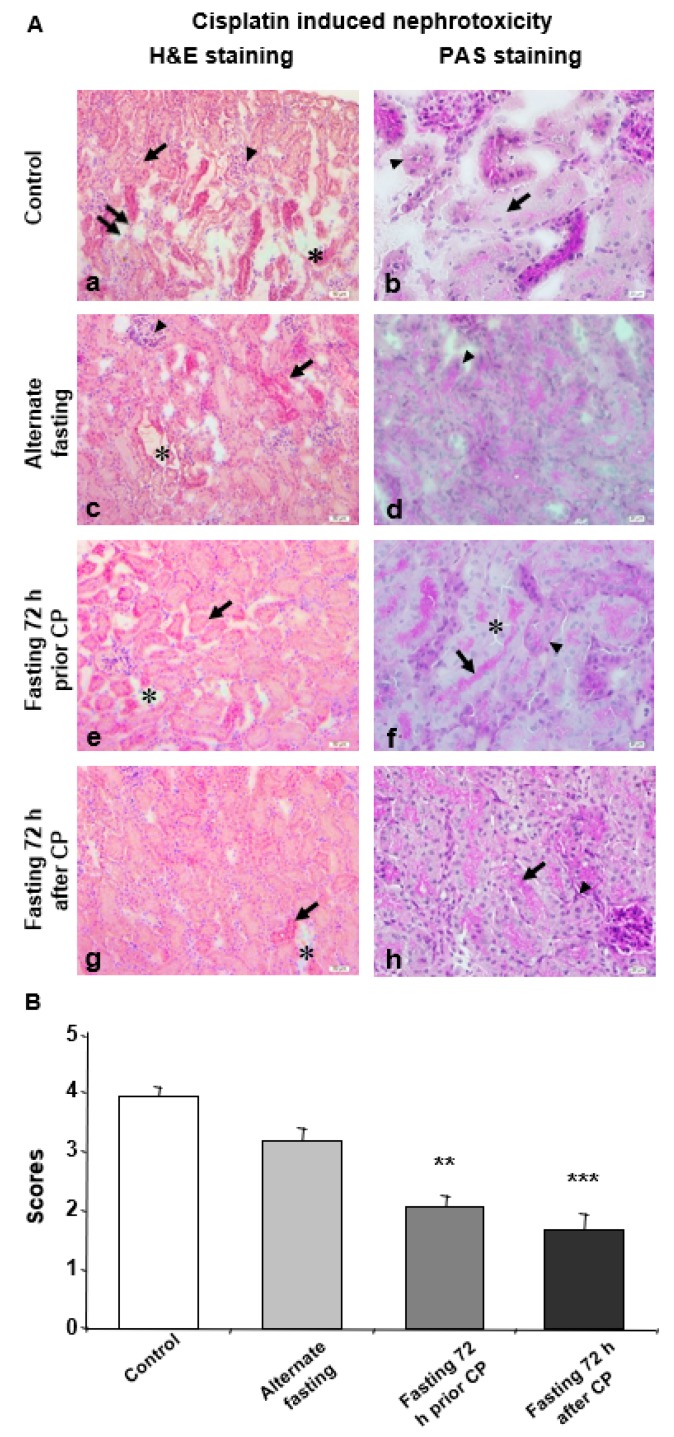
Seventy-two hours of fasting improves the histological parameters for CP induced nephrotoxicity. (**A**) Microphotographs of H&E and PAS staining. (**a**,**b**) Kidney tissue of the control group was shown. (**a**) Damaged tubules and staining difference (arrow), glomerular degeneration (arrowhead), loss of epithelial cells lining inner part of tubule (double arrow) and small gaps between the tubules (*); and (**b**) basal membrane degeneration of dysmorphic tubules (arrowhead) and degeneration in epithelial cells lining the inner part of the tubules (arrow) were demonstrated. (**c**,**d**) Kidney tissue of the alternate fasting (AF) group was shown. (**c**) Damaged tubular shape/appearance and staining difference (arrow), glomerular degeneration (arrowhead), ripped off-like gaps between the tubules (*) and (**d**) degeneration of the basal membrane (arrowhead) were demonstrated. (**e**,**f**) Kidney tissue of fasting 72 h prior CP (72P) group was shown. (**e**) Small damage and widening between the tubules (*) and tubule shape (arrow), decrease in glomerular size and (**f**) brush border at the apical surface of the epithelial cells lining the inner part of the tubule (arrow) and PAS (+) reaction of basal membranes (arrowhead). No PAS (+) reaction of basal membranes was observed at separated tubules (*) were demonstrated. (**g**,**h**) Kidney tissue of fasting 72 h after CP (72A) group was shown. (**g**) Little misshaped tubules (arrow) and gaps between neighboring tubules (*) and (**h**) brush border of tubular epithelial cells (arrow) and PAS (+) reaction of basal membranes were demonstrated (arrowhead. **a**, **c**, **e**, **g**: H&E dye, 200× magnification; **b**, **d**, **f**, **h**: PAS dye, 400× magnification. (**B**) Histological score of CP treated mice kidney in control, AF, 72P and 72A groups. Data are given as mean ± S.D. ** *p* < 0.01, and *** *p* < 0.001.

In order to examine the effect of varying fasting regimes on CPN, three DR regimes chosen, and compared with control animals which were also treated with CP but had ad libitum access to water and food. Parameters of (i) epithelial loss on the tubules, (ii) brush border loss of the remaining epithelial cells, (iii) tissue loss within intratubular space and (iv) loss of cell height were evaluated as indicators of the extent of CPN. The most extensive and severe damage was observed in the control group when compared with DR groups (Figure 1A). 

The tissue in intertubular space was more damaged and tubules were more shriveled in the control group when compared with DR groups (Figure 1A). Moreover, PAS (+) areas were decreased in the basal membranes of the deformed tubules (Figure 1A). In the AF group, this decrease, along with the severity and extent of the damage, was slightly but not significantly recovered compared to the control group (Figure 1A,B). Decreased cell height, increased brush border loss and widened intertubular spaces were detected in the AF group, together with hypertrophic but less severe glomerular degeneration (Figure 1A). Interestingly, the tubular damage inflicted by CP administration was significantly decreased in both the 72P and 72A groups compared to the control group (Figure 1A, B). Moreover, in the 72A group, epithelial cells lining the inner part of the tubules were more intact as compared with other groups. Also, the epithelial cell height was normal, and the apical brush border was present. Normal tubule appearance and diminished gaps between the tubules were also observed (Figure 1A). In the 72P group, tissue damage was decreased, and tissue integrity remained relatively intact compared to the other groups (Figure 1A). The comparison of 72A and 72P revealed decreased tissue damage in the 72P group. However, there was no statistically significant difference between the 72A and 72P groups (Figure 1B).

### 3.2. Biochemical Tests and Analysis of DNA Fragmentation Reveal that 72A Group Is Protected from CP-Induced Oxidative Stress

**Figure 2:**
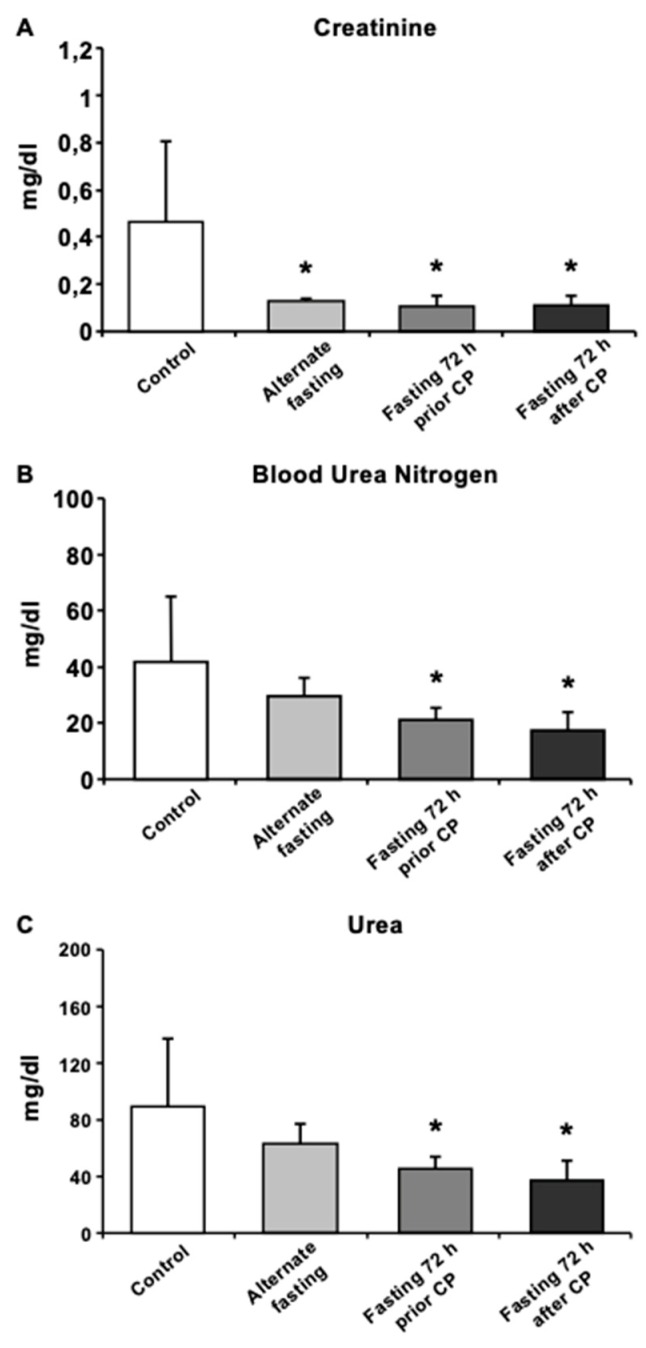
Biochemical analysis of mice serum exhibits recovery in CP induced oxidative stress and kidney function. Serum levels of (**A**) Creatinine, (**B**) BUN, (**C**) Urea were presented. Data are given as mean ± S.D. * *p* < 0.05.

To determine the CP-induced kidney functions, serum creatinine, urea and blood urea nitrogen (BUN) were assessed. In all fasting groups (AF, 72P and 72A), serum creatinine was significantly decreased (*p* < 0.05) and was highest in the control group (Figure 2A). BUN and urea levels were significantly lower in both 72 h fasting groups (72A and 72P) compared to the control (Figure 2B,C).

### 3.3. Seventy-Two Hours of Fasting Improves Cellular Survival via Regulating MAPK Pathway Elements in Kidney

**Figure 3:**
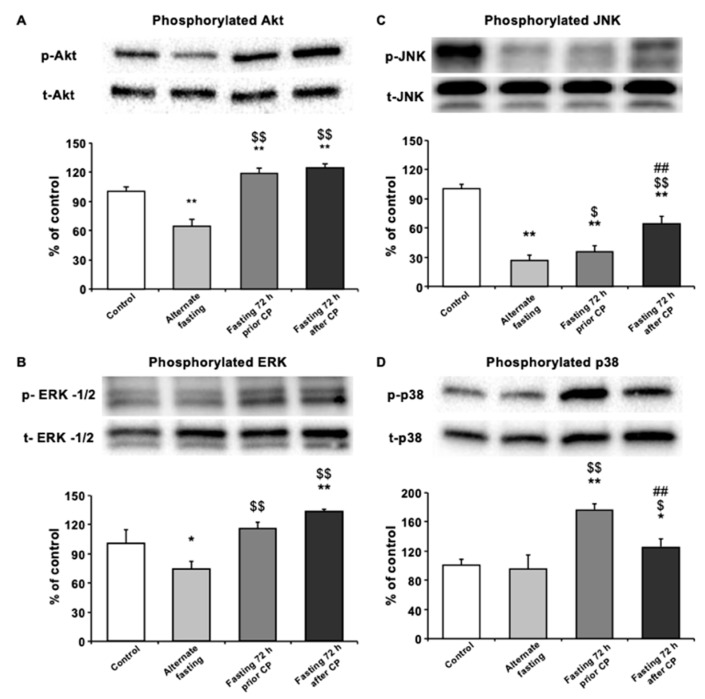
Seventy-two-hour fasting rescues kidney morphology and function through MAPK pathway. Western blot analysis of pooled tissue samples. Changes in protein levels of (**A**) p-JNK, (**B**) p-AKT, (**C**) p-ERK-1/-2 and (**D**) p-p38. * *p* < 0.05, ** *p* < 0.01 compared with control; ^$^ *p* < 0.5, ^$$^ *p* < 0.01 compared with alternate fasting; ^#^ *p* < 0.05, _##_ *p* < 0.01 compared with 72 h before.

To assess the changes in survival kinases in different dietary groups, Western blot analysis of the kidney tissues was performed. As compared with control and AF, the level of phosphorylated Akt (p-Akt), an indicator of cellular survival in kidney tissue was significantly upregulated in both 72 h DR regimes (*p* < 0.01), with no significant difference between them. Interestingly, only AF group decreased the p-Akt level compared with the control group (*p* < 0.01) (Figure 3A). In terms of another survival kinase p-ERK1/2 levels, AF group decreased p-ERK1/2 levels compared with control group, whereas both 72 h fasting groups showed an increase in p-ERK1/2 compared with AF or control (*p* < 0.01) (Figure 3B). In addition, CP administration caused a significant decrease in the phosphorylation level of c-Jun NH2-terminal kinase (JNK) in all fasting groups, compared with the control group (*p* < 0.01). On the other hand, 72P and 72A groups had significantly higher p-JNK compared to AF (*p* < 0.05 and *p* < 0.01, respectively) and compared to the other two fasting regimes, 72A group showed the highest JNK phosphorylation (both *p* < 0.01) (Figure 3C). Finally, we analyzed the phosphorylation levels of another MAPK stress signaling protein, p38 (p-p38). Results showed that, in both 72 h fasting groups, the level of p-p38 was significantly upregulated when compared with the control or AF groups (*p* < 0.05 for 72A; *p* < 0.01 for 72P) (Figure 3D). However, 72P showed a more pronounced increase in the level of p-p38 than 72A compared to control (*p* < 0.01). Finally, p-p38 level was similar to that of the control group in the AF group, following CP-induced CPN.

### 3.4. Dietary Content Is Also a Rate-Limiting Factor in Expansion of AKI Followed by CP Administration

**Figure 4:**
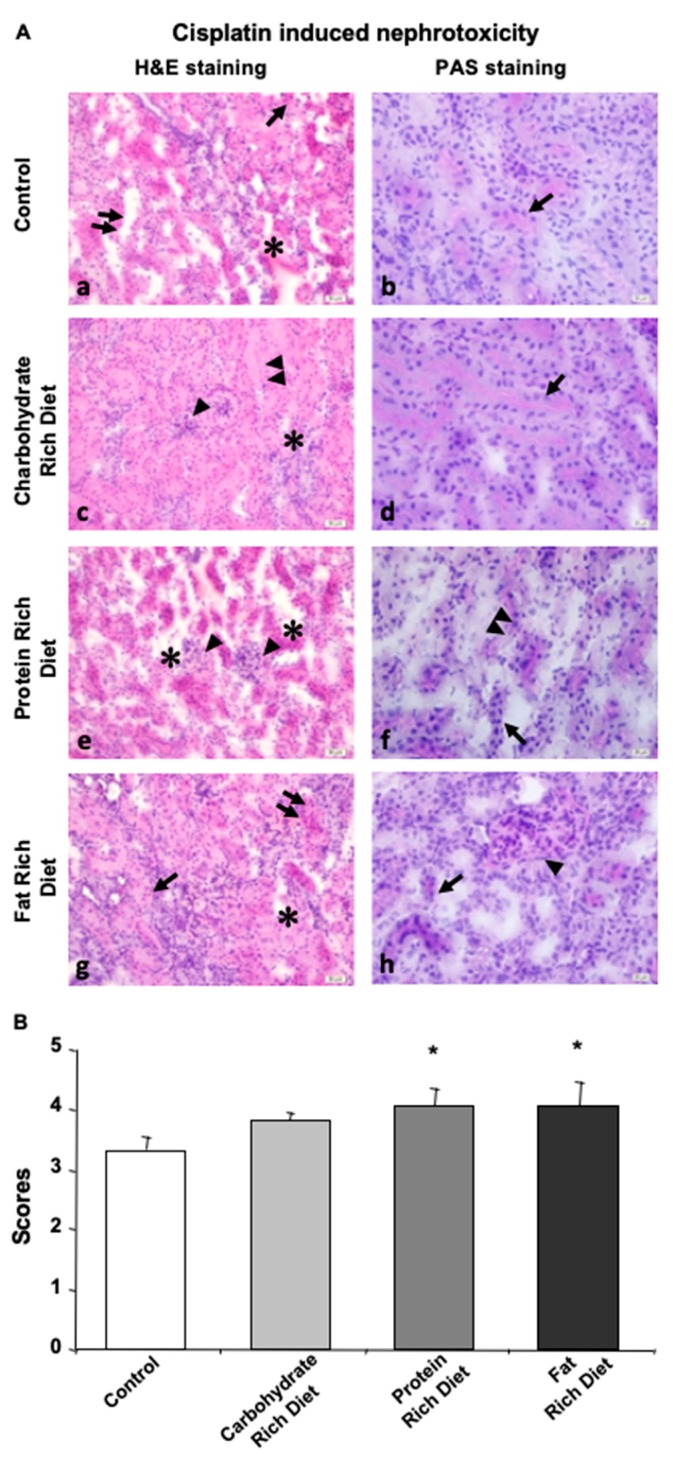
Dietary content determines the extend of CP-induced nephrotoxicity. Tissue of null diet kidney. (**A**) Microphotographs of H&E and PAS staining. (**a**,**b**) Kidney tissue of control group was shown. (**a**) Damage of tubular shape and loss of epithelial cells lining inner part of tubule (doble arrow), gaps in between the tubules (*) and inflammation (arrow), and (**b**) intact basal membrane (arrow) were demonstrated. (**c**,**d**) Kidney tissue of carbohydrate group was shown. (**c**) Dysmorphic tubules and cell loss (double arrowhead), hypertrophic glomeruli (arrowhead) and small gaps in intertubular space (*), and (**d**) basal membrane (arrow) were demonstrated. (**e**,**f**) Kidney tissue of protein group was shown. (**e**) Large separations and destructions in between the tubules (*) and loss of morphology (arrowhead) and (**f**) microvilli loss at the apical surfaces of the tubules (double arrowhead) and destruction of tubular basal membrane morphology (arrow). (**g**,**h**) Kidney tissue of fat group was shown. (**g**) Morphological disturbances and epithelial cell loss (arrow), gaps (*) and inflammation (double arrow) in between neighboring tubules and (**h**) basal membrane loss around the tubules (arrow) and glomerular dilatation (arrowhead) were demonstrated. (**a**,**c**,**e**,**g**) H&E dye, 200× magnification; (**b**,**d**,**f**,**h**) PAS dye, 400× magnification. (**B**) Histological score of CP-treated mice kidney in carbohydrate, protein, fat, or null (Control) groups were given. Data are given as mean ± SD. * *p* < 0.05.

In general, glomerular destruction and proximal- and distal-tubular cell loss was observed in all dietary groups studied dur to the injection of CP (Figure 4A,B). Nuclear dilatation and loss of microvilli on the proximal tubular cells were observed in the remaining tubular epithelial cells. In addition, dilated tubules were presented with an altered intensity in gaps and infiltration of inflammatory cells. Histopathological analysis of the study groups revealed partial epithelial loss, while some epithelial cells remained intact in distal tubules (Figure 4A, left panel). However, the basal membrane was continuous (Figure 4A, right panel). Moreover, inflammation was reduced in the control group compared to carbohydrate-, protein- and lipid-rich dietary groups.

In the carbohydrate group, glomeruli covering the urinary space were distinguishable despite being swelled. Inflammatory cell infiltration was present, through basal membranes of some tubules indicated continuity (Figure 4A).

Although intertubular gaps were observed in the control, carbohydrate and fat groups, the most enlarged gaps were detected in the protein group. There was an increase in the number of inflammatory cells and a loss in epithelial cells that form tubules and microvilli, and remaining viable cells indicated scattered and abnormal nuclei (Figure 4A). Glomerular structures were swelled (Figure 4A), and the expected PAS reaction of the basal membrane around the tubules was not observed in the protein group (Figure 4A).

The fat group displayed intensive inflammation, destroyed basal membrane of tubules, loss of tubular epithelium and enlarged nucleus, especially in the proximal tubules (Figure 4A).

Overall, these data indicated that both protein and fat groups exhibited the most severe damage while the difference that the carbohydrate diet made in renal parenchyma was not significant when compared with the control group(Figure 4B).

## 4. Discussion

CP is the most commonly used chemotherapeutic compound, and it was the first approved platinum-containing molecule by the FDA in 1978 [25]. It is stable in ambient conditions [26], and its efficacy has been shown in different types of cancers, including sarcomas, blood vessels, ovarium, testis or different kinds of solid tumors [27]. It crosslinks with the purine bases on the DNA, interferes with DNA repair mechanisms and consequently induces DNA damage in cancer cells [14]. Numerous studies have been conducted to address dose-limiting side effect of CP. It faces restrictions in clinical applications due to its side effects, such as nephrotoxicity. Even when it is applied below therapeutic concentrations, it may accumulate in the kidney tissue and cause acute irreversible kidney injury (AKI) [28].

Fasting is an attractive therapeutic approach to extend the life span of different organs and can be used as an adjuvant in the treatment of several diseases, including cancer [10]. It is estimated that healthy cells and cancer cells react to fasting differentially [19]. Nutrient deprivation inactivates proliferative signals in the cell, while it conserves protective mechanisms in turn. It has been proposed that fasting causes changes in gene expression and metabolism so that healthy cells of the body use alternate resistance pathways to prevent tissue injury. Since cancerous cells cannot exhibit such properties, chemotherapy, followed by fasting, makes those cells more vulnerable to drugs than healthy cells, possibly because of their lower ability to adapt to restricted energy supply [9,19,29].

Therefore, short-term fasting has emerged as a critical intervention for the attenuation of the side effect of chemotherapeutic compounds. In particular, 72 h short-term fasting was found feasible and was well-tolerated in a cancer patient, when DR was applied during chemotherapy. However, the role of DR in the CP-induced nephrotoxicity was still unknown [30]. Notably, it may also be a consequence of the excretion function of kidneys. In addition to the waste products of our cellular metabolism, drugs and/or their metabolites are also removed by the kidneys. In this excretion process, renal cells are exposed to such toxic molecules more than other cells in our body. In this context, here, we studied the role of short-term DR in the development of CP-induced nephrotoxicity. By using alternate day fasting (AF), 72 h fasting prior (72P) and 72 h fasting after (72A) CP treatment, we analyzed the effect of DR on CP-induced nephrotoxicity. General morphologic examination with H&E staining revealed that control group had significantly more tubular damage compared to the other groups. On the one hand, compared to the control, even with continuing hypertrophy, AF resulted in higher glomerulus integrity. On the other hand, there was a substantial decrease in structural damage in the 72P and 72A groups. Moreover, in terms of CP-induced damage extensity and severity, the control group was the most affected group. As compared with control animals, AF decreased tissue damage moderately, while 72P and 72A significantly decreased both damage extensity and severity. In general, these results indicate that fasting either 72 h prior to or after CP treatment significantly ameliorates histopathological outcomes of CP-induced nephrotoxicity.

We further analyzed the physiological outcomes of DR treatment after CP toxicity. Biochemical analysis of renal activity showed that AF significantly decreased serum creatinine levels. However, both 72 h DR regimes decreased serum creatinine, as well as BUN and urea levels. These are metabolic byproducts, and removed from the body by the kidneys. Increased levels of these molecules indicate functional failure of the kidney. In addition, these accumulated byproducts worsen kidney damage. When evaluated with histological findings, these physiological parameters suggest that fasting for 72 h after or prior to CP treatment can ameliorate AKI.

In addition, we analyzed the levels of activated survival kinases ERK and PI3K/Akt, in CP-induced nephrotoxicity. As compared with the control or AF, we observed that 72 h fasting before or after CP increases phosphorylated ERK and Akt. Interestingly, as compared with the control, the AF resulted in a decrease in the phosphorylation of these proteins. Akt phosphorylation by PI3K is essential for cellular survival and proliferation [31]. The ERK pathway is mainly regulated by an EGF Receptor/Ras/Raf signaling cascade in the oxidative-stress condition. The inhibition of ERK pathway causes cell death after kidney injury, cerebral ischemia and retinal ganglion cell injury [32,33,34]. In general, the activation of both ERK and PI3K/Akt pathways is associated with cellular survival [31].

It is interesting that signaling pathways in general or ERK and PI3K/Akt survival kinases have been a discussion of the matter with controversial results in terms of cancer cell, cellular survival and CP toxicity. It can be a consequence of different methodological approaches for the administration of pathway inhibitors and can be affected by the use of different injection coordinates of these molecules to the kidney. In a recent study, Saha et al., revealed that mangiferin ameliorates kidney injury, induced by an oxidative stress inducer, tBHP, via the deactivation of stress kinases JNK and p38 and the activation of PI3K/Akt pathways [35]. In addition, by using the PI3K inhibitor LY294002, it was shown that propofol protects kidneys via the phosphorylation of PI3K/Akt pathway after kidney injury, which was associated with the improved serum BUN level [36]. Furthermore, it was shown that CP also activates Akt pathways via EGFRi, Src and PI3K, which was associated with the activation of p38 [37].

The second downstream target of CP is JNK, and it is known to play a role in inflammation and apoptosis [38,39]. In our study, we found that JNK is significantly downregulated in all fasting groups. Recent evidence suggests that inflammation is one of the main processes that leads to proximal tubular injury [40]. Ramesh and Reeves have shown that inhibition of p38 reduces CP-induced functional and structural renal damage [41]. In our present study, we found that AF did not alter p38 phosphorylation levels compared to the control, but each of the 72 h fasting regimens increases p38 phosphorylation. Previous studies suggested that TNF-alpha inhibition by means of blocked activation of p38 provides protection from cisplatin-related renal injury and inflammation [41,42]. It can be concluded that 72 h fasting could enhance the inflammatory response against hydroxyl and other radicals and protect renal cells against CP-induced injury, which in turn may explain how 72 h fasting but not AF could ameliorate CP-induced damage in our histological findings [43]. As previously mentioned, JNK activation in both 72 h groups was significantly higher. Compared to 72A fasting 72 h fasting before CP treatment showed significantly higher p38 activation. It was reported that fasting before and after chemotherapy treatment may have different outcomes regarding injury caused by re-feeding. Long-term fasting before chemotherapy induces cell death in cancer cells, but during re-feeding, cell proliferation is induced and may lead to DNA damage in the presence of toxins [44]. 

In summary, fasting for 72 h before cisplatin treatment could prevent CP-induced kidney injury via decreased phospho-JNK and hence decreased MAPK pathway activation and caused an increase in cell cycle arrest. Furthermore, both 72 h fasting groups showed an increase in p38 MAPK activation and inflammatory response. This increase did not affect the perseverance of the kidney tubules, such that we can conclude that Akt phosphorylation may be involved in the renoprotective effects of fasting for 72 h.

As for the second part, we studied the effect of dietary contents, rather than fasting, on CP-induced nephrotoxicity. Based on histological examinations, it can be concluded that protein- and fat-rich diets caused more severe renal damage standard diet. It can be speculated that serving cancer and patients that undergo CP treatment with a low-protein and -fat diet (if not fasting) could provide beneficial effects.

Taken together, our results indicate that 72 h of fasting both prior to and after CP treatment may not only increase the efficacy of eliminating cancer cells in the organism but also ameliorates CP-induced AKI. We demonstrated that fasting-mediated rescue of renal failure takes place, at least in part, by improving renal tissue integrity and renal functions. Our findings can make a significant contribution to chemotherapeutic treatment strategies of cancer patients, by establishing a method for clinical application of CP at higher and effective doses, without causing severe AKI. 

## References

[1] von der Maase H., Sengelov L., Roberts J.T., Ricci S., Dogliotti L., Oliver T., Moore M.J., Zimmermann A., Arning M. (2005). Long-term-survival results of a randomized trial comparing gemcitabine plus cisplatin, with methotrexate, vinblastine, doxorubicin, plus cisplatin in patients with bladder cancer. J. Clin. Oncol..

[2] Silver D.P., Richardson A.L., Eklund A.C., Wang Z.C., Szallasi Z., Li Q., Juul N., Leong C.O., Calogrias D., Buraimoh A. (2010). Efficacy of neoadjuvant Cisplatin in triple-negative breast cancer. J. Clin. Oncol..

[3] Pignon J.P., Tribodet H., Scagliotti G.V., Douillard J.Y., Shepherd F.A., Stephens R.J., Dunant A., Torri V., Rosell R., Seymour L. (2008). Lung adjuvant cisplatin evaluation: a pooled analysis by the LACE Collaborative Group. J. Clin. Oncol..

[4] Miller R.P., Tadagavadi R.K., Ramesh G., Reeves W.B. (2010). Mechanisms of Cisplatin nephrotoxicity. Toxins.

[5] Oh G.S., Kim H.J., Shen A., Lee S.B., Khadka D., Pandit A., So H.S. (2014). Cisplatin-induced Kidney Dysfunction and Perspectives on Improving Treatment Strategies. Electrolyte Blood Press..

[6] Mattson M.P., Longo V.D., Harvie M. (2017). Impact of intermittent fasting on health and disease processes. Ageing Res. Rev..

[7] Estrela G.R., Wasinski F., Batista R.O., Hiyane M.I., Felizardo R.J., Cunha F., de Almeida D.C., Malheiros D.M., Camara N.O., Barros C.C. (2017). Caloric Restriction Is More Efficient than Physical Exercise to Protect from Cisplatin Nephrotoxicity via PPAR-Alpha Activation. Front Physiol..

[8] Raffaghello L., Safdie F., Bianchi G., Dorff T., Fontana L., Longo V.D. (2010). Fasting and differential chemotherapy protection in patients. Cell Cycle.

[9] Shi Y., Felley-Bosco E., Marti T.M., Orlowski K., Pruschy M., Stahel R.A. (2012). Starvation-induced activation of ATM/Chk2/p53 signaling sensitizes cancer cells to cisplatin. BMC Cancer.

[10] van Niekerk G., Hattingh S.M., Engelbrecht A.M. (2016). Enhanced Therapeutic Efficacy in Cancer Patients by Short-term Fasting: The Autophagy Connection. Front Oncol..

[11] Smith N.J.G., Caldwell J.L., van der Merwe M., Sharma S., Butawan M., Puppa M., Bloomer R.J. (2019). A Comparison of Dietary and Caloric Restriction Models on Body Composition, Physical Performance, and Metabolic Health in Young Mice. Nutrients.

[12] Descamps O., Riondel J., Ducros V., Roussel A.M. (2005). Mitochondrial production of reactive oxygen species and incidence of age-associated lymphoma in OF1 mice: effect of alternate-day fasting. Mech. Ageing Dev..

[13] Hsieh E.A., Chai C.M., Hellerstein M.K. (2005). Effects of caloric restriction on cell proliferation in several tissues in mice: role of intermittent feeding. Am J. Physiol. Endocrinol. Metab..

[14] Rocha N.S., Barbisan L.F., de Oliveira M.L., de Camargo J.L. (2002). Effects of fasting and intermittent fasting on rat hepatocarcinogenesis induced by diethylnitrosamine. Teratog. Carcinog. Mutagen..

[15] Siegel I., Liu T.L., Nepomuceno N., Gleicher N. (1988). Effects of short-term dietary restriction on survival of mammary ascites tumor-bearing rats. Cancer Invest..

[16] Wan R., Camandola S., Mattson M.P. (2003). Intermittent fasting and dietary supplementation with 2-deoxy-D-glucose improve functional and metabolic cardiovascular risk factors in rats. Faseb J..

[17] Yakar S., Leroith D., Brodt P. (2005). The role of the growth hormone/insulin-like growth factor axis in tumor growth and progression: Lessons from animal models. Cytokine Growth Factor Rev..

[18] Cheng C.W., Adams G.B., Perin L., Wei M., Zhou X., Lam B.S., Da Sacco S., Mirisola M., Quinn D.I., Dorff T.B. (2014). Pro-longed fasting reduces IGF-1/PKA to promote hematopoietic stem-cell-based regeneration and reverse immunosuppression. Cell Stem Cell.

[19] Longo V.D., Mattson M.P. (2014). Fasting: molecular mechanisms and clinical applications. Cell Metab..

[20] Jones M.M., Basinger M.A., Beaty J.A., Holscher M.A. (1991). The relative nephrotoxicity of cisplatin, cis-[Pt(NH3)2(guanosine)2] 2+, and the hydrolysis product of cisplatin in the rat. Cancer Chemother. Pharm..

[21] Saleh S., Ain-Shoka A.A., El-Demerdash E., Khalef M.M. (2009). Protective effects of the angiotensin II receptor blocker losartan on cisplatin-induced kidney injury. Chemotherapy.

[22] Chen L., Marko L., Kassmann M., Zhu Y., Wu K., Gollasch M. (2014). Role of TRPV1 channels in ischemia/reperfusion-induced acute kidney injury. PLoS ONE.

[23] Pan H., Shen K., Wang X., Meng H., Wang C., Jin B. (2014). Protective effect of metalloporphyrins against cisplatin-induced kidney injury in mice. PLoS ONE.

[24] Spandou E., Tsouchnikas I., Karkavelas G., Dounousi E., Simeonidou C., Guiba-Tziampiri O., Tsakiris D. (2006). Erythropoietin attenuates renal injury in experimental acute renal failure ischaemic/reperfusion model. Nephrol. Dial. Transpl..

[25] Kelland L. (2007). The resurgence of platinum-based cancer chemotherapy. Nat. Rev. Cancer.

[26] Karbownik A., Szalek E., Urjasz H., Gleboka A., Mierzwa E., Grzeskowiak E. (2012). The physical and chemical stability of cisplatin (Teva) in concentrate and diluted in sodium chloride 0.9%. Wspolczesna Onkol..

[27] Dasari S., Tchounwou P.B. (2014). Cisplatin in cancer therapy: Molecular mechanisms of action. Eur. J. Pharm..

[28] Peres L.A., da Cunha A.D. (2013). Acute nephrotoxicity of cisplatin: molecular mechanisms. J. Bras. Nefrol..

[29] Raffaghello L., Lee C., Safdie F.M., Wei M., Madia F., Bianchi G., Longo V.D. (2008). Starvation-dependent differential stress resistance protects normal but not cancer cells against high-dose chemotherapy. Proc. Natl. Acad. Sci. USA.

[30] Bauersfeld S.P., Kessler C.S., Wischnewsky M., Jaensch A., Steckhan N., Stange R., Kunz B., Bruckner B., Sehouli J., Michalsen A. (2018). The effects of short-term fasting on quality of life and tolerance to chemotherapy in patients with breast and ovarian cancer: a randomized crossover pilot study. BMC Cancer.

[31] Kilic U., Caglayan A.B., Beker M.C., Gunal M.Y., Caglayan B., Yalcin E., Kelestemur T., Gundogdu R.Z., Yulug B., Yilmaz B. (2017). Particular phosphorylation of PI3K/Akt on Thr308 via PDK-1 and PTEN mediates melatonin’s neuroprotective activity after focal cerebral ischemia in mice. Redox Biol..

[32] Kilic U., Kilic E., Soliz J., Bassetti C.I., Gassmann M., Hermann D.M. (2005). Erythropoietin protects from axotomy-induced degeneration of retinal ganglion cells by activating ERK-1/-2. Faseb J..

[33] Feliers D., Kasinath B.S. (2011). Erk in kidney diseases. J. Signal Transduct..

[34] di Mari J.F., Davis R., Safirstein R.L. (1999). MAPK activation determines renal epithelial cell survival during oxidative injury. Am J. Physiol..

[35] Saha S., Mahalanobish S., Dutta S., Sil P.C. (2019). Mangiferin ameliorates collateral neuropathy in tBHP induced apoptotic nephropathy by inflammation mediated kidney to brain crosstalk. Food Funct..

[36] Wei Q.J., Zhao J.J., Zhou X.G., Yu L.L., Liu Z.H., Chang Y.L. (2019). Propofol can suppress renal ischemia-reperfusion injury through the activation of PI3K/AKT/mTOR signal pathway. Gene.

[37] Winograd-Katz S.E., Levitzki A. (2006). Cisplatin induces PKB/Akt activation and p38(MAPK) phosphorylation of the EGF receptor. Oncogene.

[38] Jo S.K., Cho W.Y., Sung S.A., Kim H.K., Won N.H. (2005). MEK inhibitor, U0126, attenuates cisplatin-induced renal injury by decreasing inflammation and apoptosis. Kidney Int..

[39] Mukherjee S., Dash S., Lohitesh K., Chowdhury R. (2017). The dynamic role of autophagy and MAPK signaling in determining cell fate under cisplatin stress in osteosarcoma cells. PLoS ONE.

[40] Sen Z., Jie M., Jingzhi Y., Dongjie W., Dongming Z., Xiaoguang C. (2017). Total Coumarins from Hydrangea paniculata Protect against Cisplatin-Induced Acute Kidney Damage in Mice by Suppressing Renal Inflammation and Apoptosis. Evid. Based Complement Altern. Med..

[41] Ramesh G., Reeves W.B. (2005). p38 MAP kinase inhibition ameliorates cisplatin nephrotoxicity in mice. Am J. Physiol. Ren. Physiol..

[42] Mishima K., Baba A., Matsuo M., Itoh Y., Oishi R. (2006). Protective effect of cyclic AMP against cisplatin-induced nephrotoxicity. Free Radic. Biol. Med..

[43] Quintanilha J.C.F., de Sousa V.M., Visacri M.B., Amaral L.S., Santos R.M.M., Zambrano T., Salazar L.A., Moriel P. (2017). Involvement of cytochrome P450 in cisplatin treatment: implications for toxicity. Cancer Chemother. Pharm..

[44] Cangemi A., Fanale D., Rinaldi G., Bazan V., Galvano A., Perez A., Barraco N., Massihnia D., Castiglia M., Vieni S. (2016). Dietary restriction: could it be considered as speed bump on tumor progression road?. Tumour. Biol..

